# Experimental Investigation on Passive Survivability of Lithium-Ion Batteries Under Extremely Low Temperatures

**DOI:** 10.3390/s25041160

**Published:** 2025-02-14

**Authors:** Ali Soleimani Borujerdi, Jinying Zhu, Bo Zhang, Yinsheng Guo

**Affiliations:** 1 Department of Civil and Environmental Engineering, University of Nebraska-Lincoln, Omaha, NE 68182, USA; asoleimaniborujerd2@huskers.unl.edu; 2 Department of Chemistry, University of Nebraska-Lincoln, Lincoln, NE 68583, USA; bzhang26@unl.edu (B.Z.); yinshengguo@unl.edu (Y.G.)

**Keywords:** extremely low temperatures, Li-ion battery, ultrasonic testing, amplitude, dry ice, liquid nitrogen

## Abstract

This paper presents an experimental study using an ultrasonic technique to investigate the impact of extremely low temperatures on the performance of lithium-ion batteries. Lithium-ion polymer batteries were aged in three low-temperature conditions: in a temperature chamber (−34 °C), in a dry ice bath (−78 °C), and in a liquid nitrogen bath (−196 °C). The battery aged in liquid nitrogen was damaged. The batteries aged in the chamber and dry ice bath were then subjected to charge and discharge cycles and simultaneously monitored using the ultrasonic technique. Three key ultrasonic parameters were measured, signal amplitude, time of flight (TOF), and TOF shift, using 5 MHz commercial ultrasonic transducers. These measurements were conducted alongside electrical measurements (voltage and current) to monitor the batteries throughout the testing cycles. The results showed that the aged batteries exhibited significantly lower ultrasonic amplitude compared to the control batteries. Additionally, as the cycle number increased, the TOF increased and the discharge capacity decreased. The TOF shift increased linearly with the discharge capacity. However, no clear correlation was observed between the slope of this linear relationship and the low-temperature aging history of the batteries. Overall, the ultrasonic amplitude proved to be a reliable parameter for differentiating the control and low-temperature aged batteries.

## 1. Introduction

New applications of lithium-ion batteries in space exploration require them to perform effectively in extremely low temperatures [1]. Some studies have investigated the electrical and electrochemical properties of lithium-ion batteries under such conditions [1,2,3,4,5,6,7]. Nagasubramanian [2] showed that battery performance significantly declined at subfreezing temperatures in terms of power and energy. Using the two- and three-electrode impedance measurements, the study identified that the poor performance could be attributed to the high impedance of the cathode–electrolyte interface [2]. Another study by Ji et al. [3] investigated the effect of low temperatures, using temperatures as low as −20 °C, through both experiments and modeling. The study found that battery self-heating notably affects electrochemical performance, with poor performance being linked to the diffusion of Li^+^ ion and solid-state lithium in the electrolyte and graphite particles, respectively [3]. Zhu et al. [4] recommended enhancing lithium-ion battery performance at low temperatures by providing a pathway for the quick transport of Li^+^ ions and electrons.

X-ray and neutron scattering techniques have been used to detect physical changes in batteries [8]. However, these methods are expensive and difficult to implement [8]. Recently, ultrasonic techniques have been used to monitor the behavior of lithium-ion batteries at room temperature [8,9,10,11,12]. These techniques provide insights into changes in the material density and stiffness of batteries during charge and discharge cycling. Other studies on rechargeable batteries, such as zinc-ion batteries, have explored how covalent organic frameworks (COFs) improve material stability under extreme conditions, which may offer useful insights for lithium-ion batteries as well [13]. Key ultrasonic parameters, including signal amplitude, time of flight (TOF), and TOF shift, can be monitored alongside cycling parameters (e.g., voltage and current) during testing. The TOF shift is the change in the TOF between a received signal and the reference signal. These parameters could be continuously monitored during electrical cycling after batteries experience extremely low temperature conditions.

In this paper, three groups of Li-ion polymer batteries were first passively aged under extremely low-temperature conditions and then tested and monitored during charge/discharge cycles. The batteries were subjected to three low-temperature cycling conditions: (1) in a temperature chamber (+34 °C–−34 °C); (2) in a dry ice bath (−78 °C–+23 °C); (3) in a liquid nitrogen bath (−196 °C–+23 °C). Batteries of two different capacities were tested (750 mAh and 1050 mAh). This study aims to investigate the feasibility of using ultrasonic waves to evaluate battery deterioration after exposure to extremely low temperatures.

## 2. Experimental Procedure

### 2.1. Specifications of the Batteries

Lithium-ion polymer batteries with a ${\mathrm{LiCoO}}_{2}$ cathode and a graphite anode were used in this study. Batteries with two capacities were tested: 750 mAh and 1050 mAh. The dimensions were 52 × 30.5 × 6.2 mm for the 750 mAh batteries and 67 × 35.5 × 6.3 mm for the 1050 mAh batteries. The following nomenclature was used to label the batteries: aging (G) group-capacity and control (C)-capacity. The first symbol denotes the aged specimen group as follows: G1 for the temperature chamber; G2 for the dry ice bath; G3 for the liquid nitrogen bath. The following number specifies the capacity of the battery (either 750 mAh or 1050 mAh). Cycling tests were conducted on the aged batteries, with a corresponding control battery tested alongside each aged battery. The control batteries were named in the same way as the aged batteries. Table 1 lists the batteries tested in this study.

### 2.2. Low-Temperature Conditioning Protocols

The batteries were conditioned using three low-temperature methods: a temperature chamber (group 1), a dry ice bath (group 2), and a liquid nitrogen bath (group 3). In each group, one battery was subjected to the temperature cycles, and one battery was stored at room temperature as a control sample. The test procedures for different conditioning groups are discussed in the following sections.

#### 2.2.1. Group 1: Temperature Chamber

**Figure 1:**
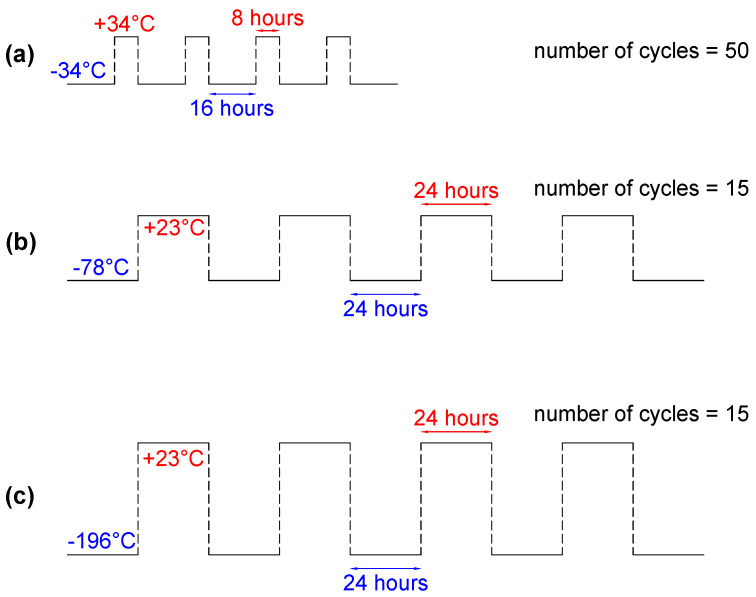
Low-temperature aging protocols: (**a**) temperature chamber: −34∼+34 °C; (**b**) dry ice bath: −78∼+23 °C; (**c**) liquid nitrogen: −196∼+23 °C.

A CSZ Z-Plus environmental test chamber was used to cycle the batteries between −34 °C and +34 °C. Two batteries (750 mAh and 1050 mAh) were conditioned for 50 cycles. Each cycle included four steps as follows: (1) a −34 °C temperature for 16 h; (2) a 15 min rest; (3) a +34 °C temperature for 8 h; (4) a 15 min rest. Figure 1a shows the schematic temperature protocol.

#### 2.2.2. Group 2: Dry Ice Bath

In the second group, one 750 mAh battery and one 1050 mAh battery underwent 15 thermal cycles using the dry ice bath method. Each cycle consisted of 24 h in a dry ice bath at −78 °C, followed by 24 h at room temperature (around 23 °C) for a total duration of 30 days. There was a 15 min rest between each step. Figure 1b shows the temperature protocol for this method.

#### 2.2.3. Group 3: Liquid Nitrogen Bath

In the third test group, liquid nitrogen was used to create an extremely low-temperature environment for the batteries. Each cycle consisted of 24 h at −196 °C, followed by 24 h at room temperature (approximately 23 °C), over a total period of 30 days. A period of 15 min of rest was used between each step. Figure 1c shows the temperature protocol for this method.

### 2.3. Charge/Discharge Cycling

**Figure 2:**
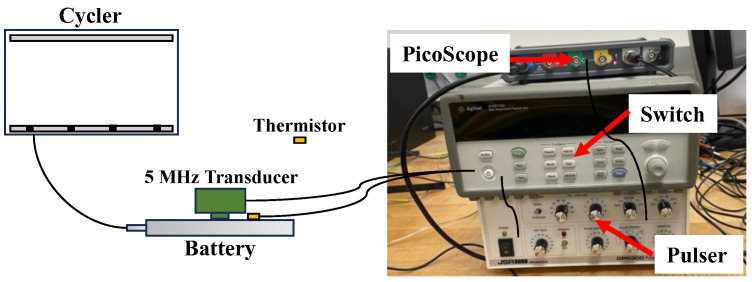
Ultrasonic monitoring test setup.

After low-temperature conditioning, the aged and companion control batteries were tested in charging and discharging cycles using a Neware BTS-3000 battery cycler (Figure 2). The batteries were charged and discharged with three different C-rates: 0.1, 0.5, and 1 C. The battery cycling protocol for each C-rate was as follows: first, the batteries were charged in a constant current (CC) mode up to 4.2 V, then they continued to charge in constant voltage (CV) mode until the current reached the cutoff current of 0.05 C, and finally they were discharged in the CC mode until the cutoff voltage of 2.75 V was reached. A 10-min rest period was used between each charge and discharge cycle. The batteries first went through a short-term cycling test (6 cycles), followed by a long-term cycling test (more that 200 cycles). The battery in Group 3 (liquid nitrogen bath) was damaged in the low-temperature aging program and therefore was not tested in the cycling phase. Table 1 shows the battery tested in each temperature conditioning and cycling group.

### 2.4. Ultrasonic Test

**Figure 3:**
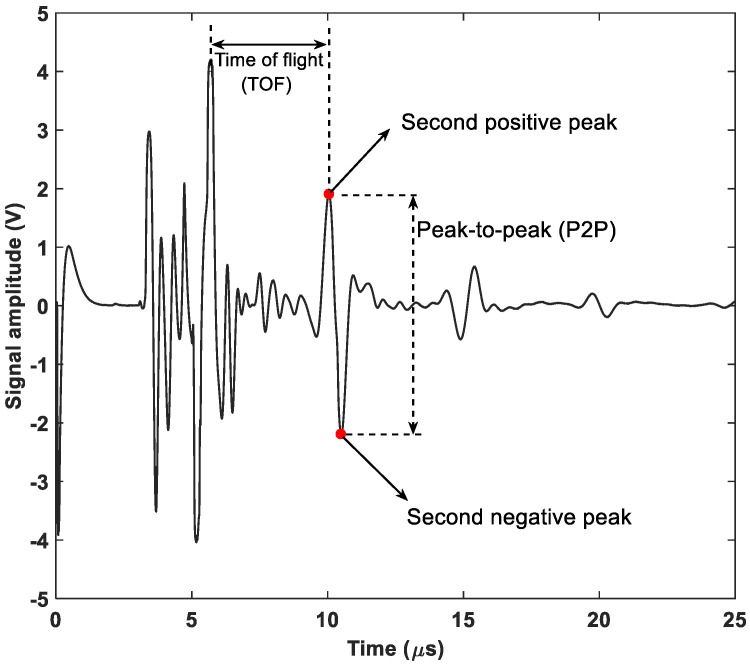
A typical ultrasonic signal.

During the cycling test, all batteries were monitored using the ultrasonic test. Figure 2 shows the ultrasonic test setup. The batteries were tested in pulse-echo mode in the thickness direction using a 5 MHz (Olympus V109) transducer. A DPR300 pulser/receiver was used for sending and receiving the ultrasonic signals. The transducer was clamped to the center of the battery and a layer of petroleum gel was used as a couplant between the battery and the transducer. The ultrasonic signals were acquired using a PicoScope (5242A) at a sampling rate of 250 MHz, and the signals were recorded every 20 s. Each test also included a control battery that was not subjected to low-temperature conditioning. To test two batteries at the same time, an Agilent 34970A switch unit was used to switch the pulser-receiver and the Picoscope devices. A LabVIEW program was developed to automate the testing and data acquisition process. Figure 3 shows a typical ultrasonic signal. The first echo was mixed with the transducer surface echo, so the second echo was selected for signal analysis. The peak-to-peak (P2P) amplitude was defined as the difference between the positive and negative peaks.

### 2.5. Temperature Effect Correction

The ultrasonic amplitude and TOF are significantly affected by temperature [14,15,16]. With increase in temperature, the signal amplitude tends to decrease, and the wave travel time TOF increases. The temperature effects should be corrected in order to use ultrasonic parameters for the characterization of battery properties. In this paper, we used the in situ temperature effect correction method proposed by Borujerdi et al. [14] to reduce the temperature effect on the TOF and TOF shift, which was defined as the change in TOF relative to a reference signal. During the cycling test, at each state of charge (SOC) of the batteries, the TOF shift and temperature data for that specific SOC were extracted and plotted. It was found there was good relationship between the TOF shift and temperature at each SOC, and this relationship only changed slightly for different SOCs. Therefore, the linear fit of the TOF shift and temperature data could be used for temperature correction. The results for the temperature-corrected TOF shift will be discussed in the following sections.

## 3. Test Results and Discussion

### 3.1. Physical Observations

**Figure 4:**
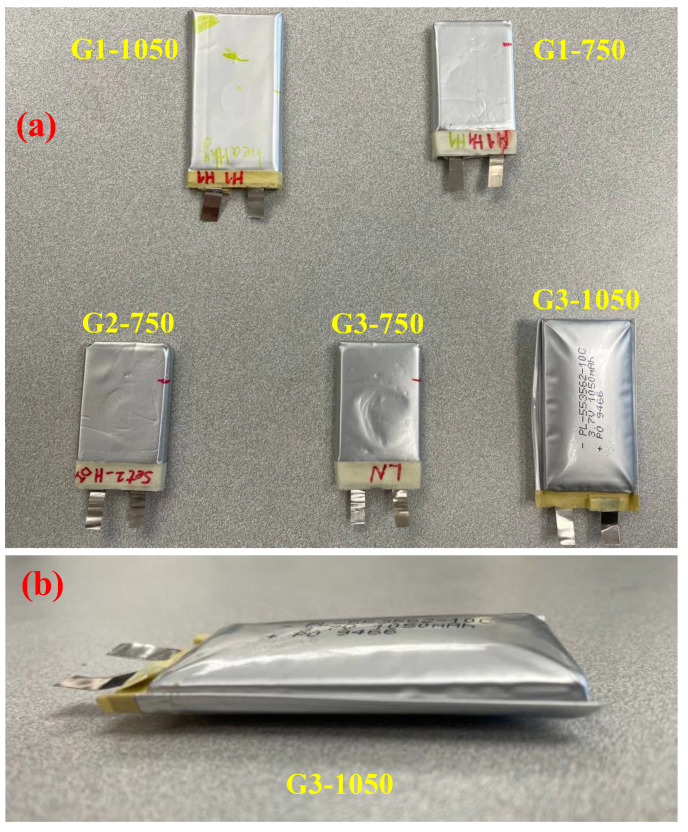
Physical observations of the batteries after aging: (**a**) 5 aged batteries; (**b**) G3-1050 from a closer look.

Figure 4 shows the batteries after thermal cycles. No liquid leakage was observed in all batteries after testing. Thermal cycle in the temperature chamber did not affect the physical state of the battery (G1-750 and G1-1050 in Figure 4). For the battery in the dry ice bath (G2-750 in Figure 4a), the surface of the battery became softer after low-temperature aging. The battery was still testable, and we could obtain reliable ultrasonic signals from the battery.

The 1050 mAh battery (G3-1050) exhibited significant swelling after being placed in a liquid nitrogen bath (Figure 4b). Similar swelling behavior has been reported in lithium-ion batteries exposed to low temperatures in [17], where electrode deformation, lithium dendrite formation, and material delamination were identified as key contributors. Lithium plating and structural stress may have further exacerbated the expansion. Due to the observed swelling and potential internal damage, cycling and ultrasonic tests were not performed for safety reasons. Meanwhile, the 750 mAh battery (G3-750) lost surface rigidity without noticeable swelling. Although a voltage of 3.6 V was still present, no measurable ultrasonic echo signal was obtained, suggesting possible internal structural changes affecting wave propagation.

### 3.2. Short-Term Monitoring

Both short-term and long-term cycling tests were performed on the batteries aged in the temperature chamber (group 1) and dry ice bath (group 2), along with ultrasonic monitoring. In the short-term cycling test, the batteries were tested for six cycles. To examine the long-term performances of the batteries, they were tested for an extended 300 (group 1) and 200 (group 2) cycles. The short-term monitoring results are presented first.

#### 3.2.1. Group 1: Specimens in Temperature Chamber

**Figure 5:**
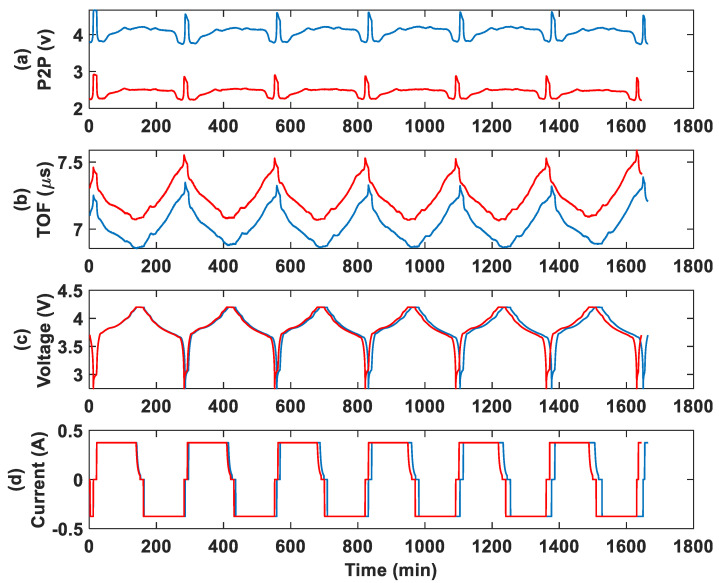
Group 1 cycling test results at a 0.5 C rate for the control (blue) and chamber aged (red) 750 mAh batteries: (**a**) P2P amplitude, (**b**) TOF, (**c**) voltage, and (**d**) current.

The ultrasonic and cycling results for the 750 mAh battery at 0.5 C are shown in Figure 5, which includes the ultrasonic P2P amplitude, the TOF, and the cycling voltage and current for both control and aged batteries. A repeatable trend is observed in the ultrasonic parameters for both control and aged batteries. During the charging process, the P2P amplitude increases, while the TOF decreases. In contrast, during discharging, the P2P amplitude decreases, and the TOF increases. These changes in ultrasonic amplitude and wave velocity are associated with variations in the material density of the battery and the elastic modulus of the anode material (i.e., graphite) [9] during cycling.

According to previous studies [9,18,19], the influence of graphite on wave velocity is more pronounced than that of lithium cobalt oxide (LCO) due to the distinct material property variations during charge and discharge. The stiffness of graphite decreases by a factor greater than three during discharge, while LCO stiffens by a factor of two. In addition, LCO’s stiffness remains relatively constant until its degree of lithiation exceeds 0.9, which occurs primarily at the very end of discharge. Since ultrasonic wave propagation speed is directly linked to material stiffness, these variations contribute to the observed differences in wave velocity. Additionally, graphite has a lower volumetric specific capacity than LCO [20], meaning that it occupies a larger volume fraction within the cell, thereby exerting a more substantial influence on the overall wave propagation time and TOF. Consequently, the graphite anode plays a dominant role in determining wave velocity trends during cycling, explaining why changes in wave velocity are more strongly expressed in graphite than in lithium cobalt oxide.

A spike is observed in the P2P amplitude curve near full discharge, which could be attributed to a structural transformation within the battery [9]. Similar observations have been reported in previous studies. Hsieh et al. [8] identified a sharp increase in signal amplitude near the end of discharge, linking it to a hexagonal-to-monoclinic phase transition in ${\mathrm{LiCoO}}_{2}$, which significantly alters both the modulus and density of the cathode material. Davies et al. [9] also reported a significant and rapid change in ToF shift and signal amplitude at a very low SOC, suggesting that substantial structural changes occur at this stage, potentially serving as an indicator of over-discharge. In another study by the authors [14], we also observed this spike feature in batteries with different capacities at different cycling rates. The consistency of these observations across different studies and battery conditions indicates that this phenomenon is not an anomaly but rather an intrinsic aspect of the electrochemical and mechanical behavior of the active materials near full discharge.

It is clearly observed that the P2P amplitude for the aged battery is significantly lower than that of the control battery (Figure 5a), which may indicate the initiation of damage due to the low-temperature aging conditions. Additionally, the TOF for the aged battery is noticeably higher than that of the control. By analyzing both ultrasonic parameters (P2P and TOF), we can effectively differentiate the aged battery from the control battery.

From the voltage and current curve in Figure 5, we also notice that the cycling duration becomes shorter with each cycle. Given the same cycling protocol, this shorter duration suggests a reduction in battery capacity with increasing cycle numbers. This phenomenon becomes even more pronounced in the long-term cycling tests, which will be discussed later.

#### 3.2.2. Group 2: Specimens in Dry Ice

**Figure 6:**
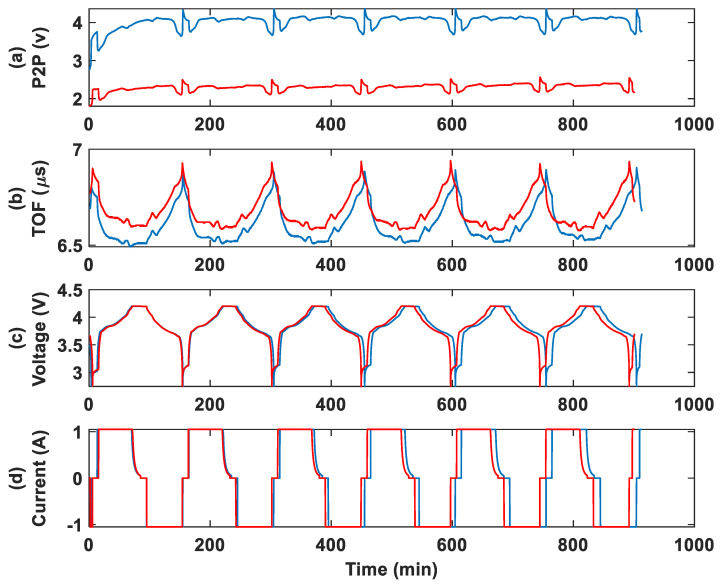
Group 2 cycling test results at 1 C rate for the control (blue) and dry ice bath aged (red) 1050 mAh batteries: (**a**) P2P amplitude, (**b**) TOF, (**c**) voltage, (**d**) and current.

This section shows the results of the ultrasonic monitoring of the group 2 batteries. Figure 6 shows the ultrasonic and cycling results for the control and aged batteries with 1050 mAh capacity. The aged battery is placed in a dry ice bath, as detailed in the experimental procedure section. The first observation is a noticeable drop in the P2P amplitude for the aged battery compared to the control. In the TOF trend, a deviation from the control is observed in the aged battery. This difference appears to increase further during long-term monitoring. The cycle duration for a full charge/discharge cycle of the aged battery begins to decrease in the last three cycles, indicating a reduction in capacity.

### 3.3. Long-Term Monitoring

**Figure 7:**
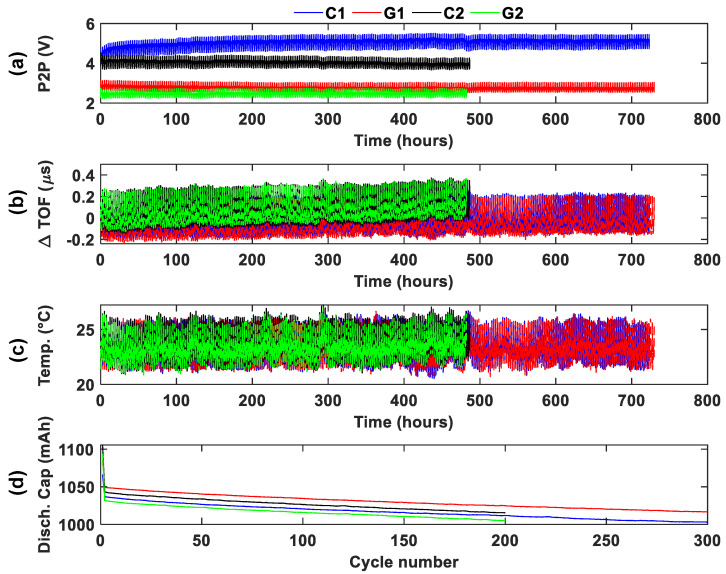
Ultrasonic results for long-term monitoring of 1050 mAh battery at 1C (groups 1 and 2): (**a**) P2P signal amplitude, (**b**) TOF shift, (**c**) temperature, (**d**) and discharge capacity.

To better understand the behavior of batteries over a long-term period, the control and aged batteries in groups 1 and 2 were tested for an extended duration of 300 and 200 cycles, respectively, following short-term monitoring. These tests were conducted on the control and aged 1050 mAh batteries (Table 1). Figure 7 presents the ultrasonic amplitude, TOF shift, temperature, and discharge capacity results for all the batteries tested in group 1 and group 2. The results are shown in a single figure to facilitate comparison.

Unlike in the short-term cycling test, Figure 7d shows that low-temperature conditioning did not have a significant effect on the discharge capacity of the batteries. All batteries, including the control ones, show a stable capacity fade at a similar rate, indicating that low-temperature aging did not significantly affect electrochemical performance. However, the ultrasonic P2P amplitude shows a significant drop between the control and aged batteries. The aged batteries in both groups exhibit much lower P2P amplitudes compared to the control, suggesting that low-temperature aging caused the deterioration of the internal physical structure of the batteries.

The TOF shift trend exhibits a steady upward progression throughout the cycling period for all batteries. However, the distinction between control and aged batteries in terms of TOF shift is not immediately apparent when examining the entire cycling period in a single plot. A more detailed analysis, focusing on specific cycle segments, will be presented later to highlight these differences.

Lastly, the temperature data show limited variation during cycling, with a trend similar to the TOF shift. This trend indicates the temperature effect on the TOF shift, which will be discussed in the next section.

### 3.4. Temperature Correction

As previously discussed, we used the correlation between the TOF shift and temperature at different SOC levels to reduce the effect of temperature. As discussed in [14], the temperature correction factor typically falls within the range of 0.03 to 0.05 μs/°C. Once the TOF is fully corrected for temperature effect, the TOF curve will show minimal correlation with temperature and gives a smooth progression with the cycle number. In this study, we tested different correction factors and found that a value of 0.04 provides the best results for temperature correction. After temperature correction, the TOF shift reveals more detailed distinctions between control and aged batteries compared to the absolute TOF values.

**Figure 8:**
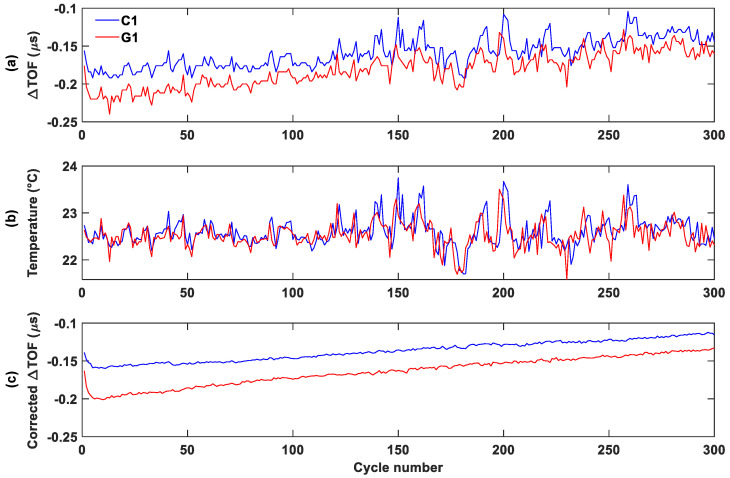
Group 1 temperature-correction process for the control (blue) and aged (red) batteries: (**a**) uncorrected minimum TOF shift, (**b**) temperature change, (**c**) and corrected minimum TOF shift.

**Figure 9:**
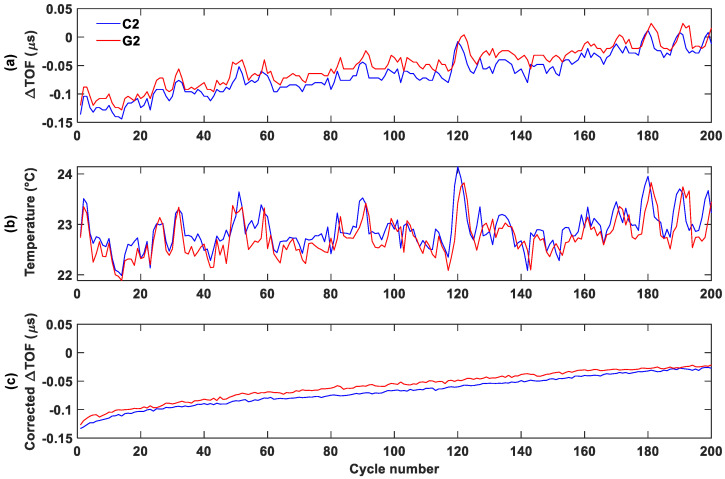
Group 2 temperature-correction process for the control (blue) and aged (red) batteries: (**a**) uncorrected minimum TOF shift, (**b**) temperature change, (**c**) and corrected minimum TOF shift.

To better visualize the TOF shift trend, the minimum TOF shift, which approximately corresponds to the 100% SOC for each cycle, was plotted against the cycle number both before and after temperature correction (Figure 8 and Figure 9). Before temperature correction, the TOF shift exhibited significant noise. After applying correction using a 0.04 correction factor, all batteries showed a much smoother TOF curve.

### 3.5. TOF Shift Analysis Across Battery Groups

**Figure 10:**
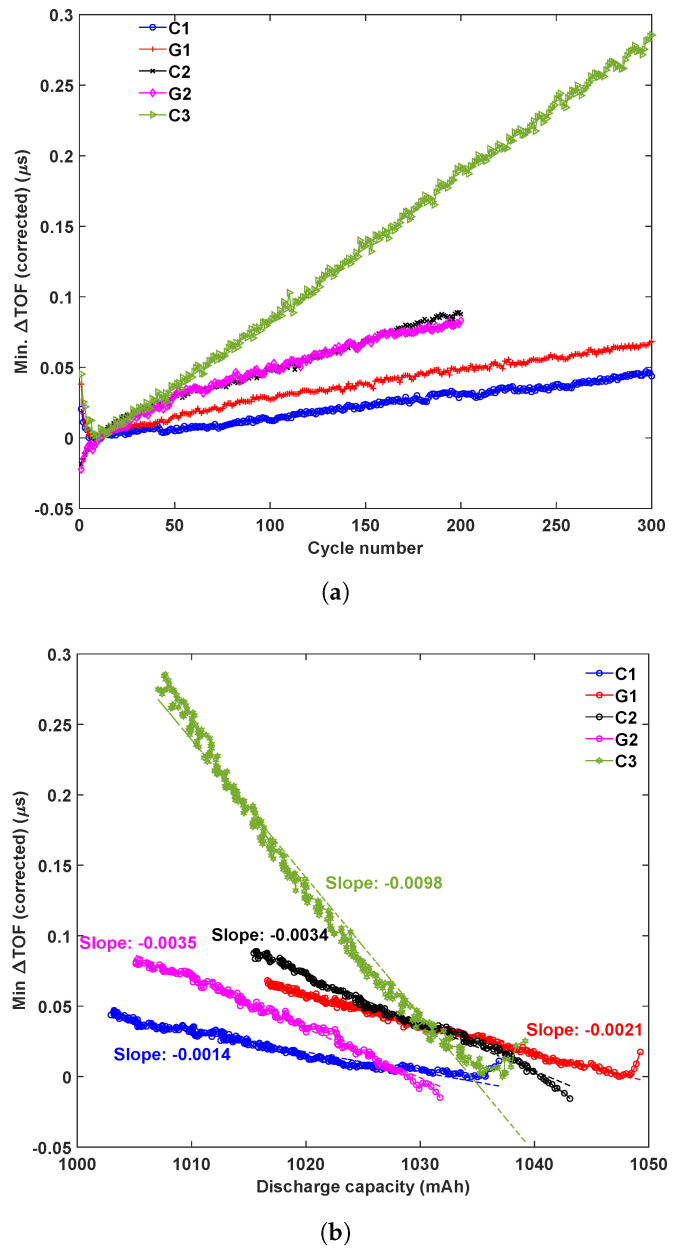
Temperature-corrected minimum TOF shift change with (**a**) cycle numbers and (**b**) discharge capacities for all batteries.

To compare the results of all batteries, the minimum TOF shift is plotted against the cycle number in Figure 10a. Because the results in the first few cycles were unstable, for each battery, we use the minimum TOF shift of the 10th cycle as the reference. For all batteries, the TOF shift increases almost linearly with the cycle number, which indicates decreases in the ultrasonic wave velocity and elastic modulus of the batteries. However, there is no clear trend between the slopes of the relationship and battery aging conditions. For group 1, the aged battery shows a slightly larger slope than the control, while for group 2, both the control and aged batteries show a similar trend. The two control batteries in group 1 and group 2 show very different slopes. It is unclear if this discrepancy in the control batteries is related to the initial structure of the batteries or the cycling program. To answer this question, we include the cycling data from another control battery C3 (1050 mAh), which was tested at a 1.0 C rate in a separate test. It is shown that this control battery also shows a linear relationship between the TOF shift and the cycle number, but with a very different slope. From these limited number of tests, the authors believe that the cycling program and test conditions may have a large influence on the TOF shift trend with cycle numbers, because the difference between different test groups is much larger than the difference between the aged and control batteries in the same test group.

Figure 10b shows the minimum TOF shift variation with the discharge capacity during the long-term cycling tests. There is a linear relationship between the minimum TOF shift and discharge capacity for each battery, and the TOF shift increases with decreasing capacity. However, similar to Figure 10a, we do not see a clear pattern to differentiate the control and aged batteries from the TOF shift vs. capacity relationships.

### 3.6. Ultrasonic Amplitude Change

**Figure 11:**
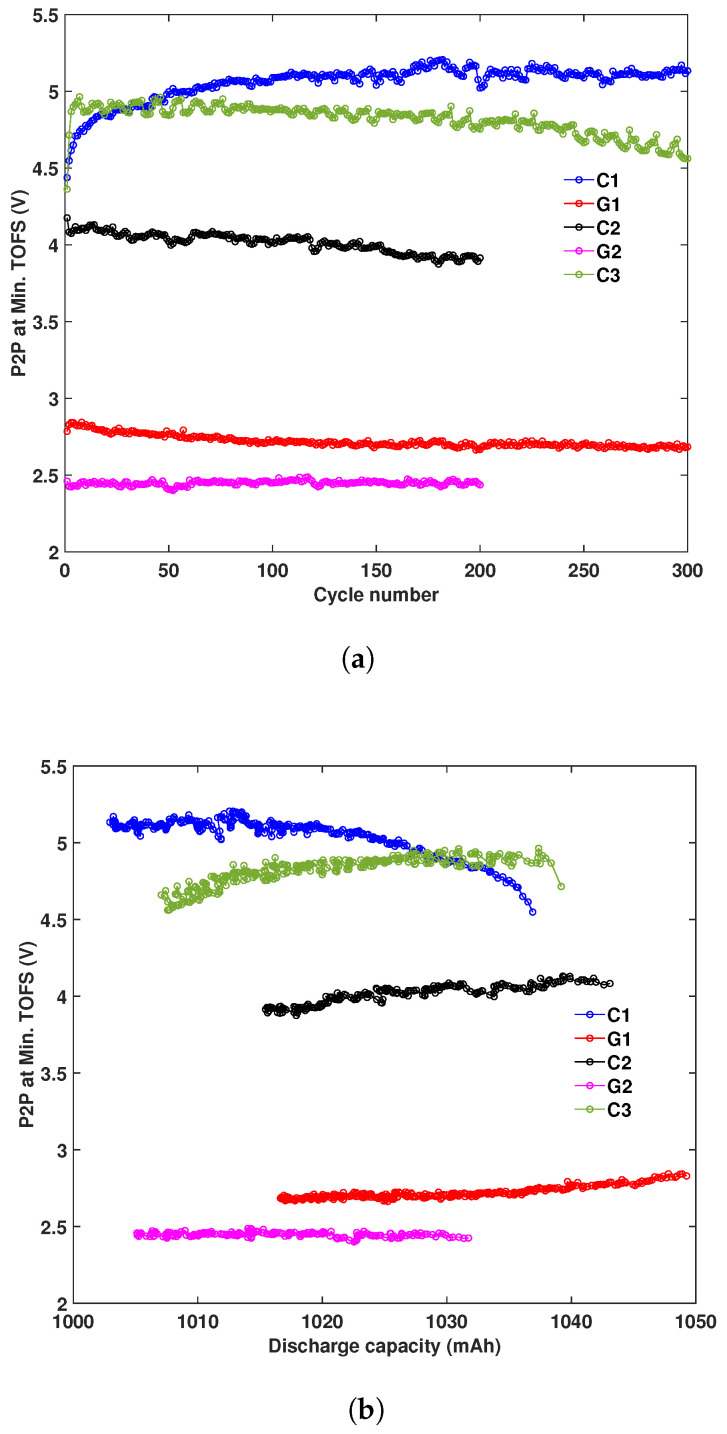
Ultrasonic amplitude change with (**a**) cycle numbers and (**b**) discharge capacities for all batteries.

Figure 11 presents the changes in ultrasonic amplitude with cycle numbers and discharge capacity for all batteries. The P2P amplitude corresponds to the minimum TOF shift point in each cycle. It is seen that the three control batteries exhibit significantly higher amplitudes compared to the aged batteries. For batteries C2, C3, and G1, the ultrasonic amplitudes decrease as the cycle number increases and the discharge capacity decreases. The amplitude for battery G2 remains nearly constant throughout the cycling process, while battery C1 shows an increasing trend with the cycle number.

## 4. Conclusions

This study investigated the effect of extremely low temperatures on the aging of lithium-ion batteries using the ultrasonic technique. Batteries in three test groups were cyclically conditioned in three different low-temperature environments: a temperature chamber (−34∼+34 °C), dry ice bath (−78∼+23 °C), and liquid nitrogen bath (−196∼+23 °C). In each group, one control battery was kept at room temperature as a reference. After conditioning, the batteries were tested using a battery cycler and monitored by ultrasonic waves. Based on the results of this study, the following conclusions could be drawn:

1. The batteries conditioned in the chamber and dry ice bath did not show a significant capacity drop. The aged and control batteries had similar discharge capacities, close to the nominal capacity.
2. The batteries in the liquid nitrogen bath were not usable and testable after conditioning.
3. The TOF (or TOF shift) changed linearly with the charge/discharge cycle number. This finding may be used to evaluate cycling aging based on TOF. However, we could not reach a clear conclusion on how low-temperature aging affects the TOF value or the TOF shift vs. cycle relationship. Further study is needed to understand the physical property changes in batteries aged in low-temperature conditions and the effects on their long-term performance.
4. For the batteries aged in the temperature chamber and dry ice bath, their ultrasonic amplitudes were much smaller compared to those of the control batteries. Although the electrical capacity was not significantly affected by low-temperature aging, the reduced ultrasonic amplitudes indicate physical changes in the internal structures of the batteries.
5. Based on the data presented in this study, the ultrasonic amplitude was a reliable parameter that clearly differentiated the control and low-temperature aged batteries, even though the discharge capacity was not significantly affected by aging.

## Figures and Tables

**Table 1:** Control and aged batteries tested in this study.

Aging Method	Chamber		Dry Ice Bath			Liquid Nitrogen Bath
**Cycling**	**Short-Term**	**Long-Term**	**Short-Term**	**Long-Term**	**No Cycling**	**No Cycling**
Control	C1-750	C1-1050	C2-1050	C2-1050	—	—
Aged	G1-750	G1-1050	G2-1050	G2-1050	G2-750	G3-750, G3-1050

## Data Availability

Data will be available upon reasonable request.

## Abbreviations

The following abbreviations are used in this manuscript:

TOF	Time-of-flight
P2P	Peak-to-peak amplitude
SOC	State of charge

## References

[1] Nandini K., Usha K., Srinivasan M., Pramod M., Satyanarayana P., Sankaran M. (2018). Study on survivability of 18650 Lithium-ion cells at cryogenic temperatures. J. Energy Storage.

[2] Nagasubramanian G. (2001). Electrical characteristics of 18650 Li-ion cells at low temperatures. J. Appl. Electrochem..

[3] Ji Y., Zhang Y., Wang C.Y. (2013). Li-ion cell operation at low temperatures. J. Electrochem. Soc..

[4] Zhu G., Wen K., Lv W., Zhou X., Liang Y., Yang F., Chen Z., Zou M., Li J., Zhang Y. (2015). Materials insights into low-temperature performances of lithium-ion batteries. J. Power Sources.

[5] Aris A.M., Shabani B. (2017). An experimental study of a lithium ion cell operation at low temperature conditions. Energy Procedia.

[6] Grandjean T.R., Groenewald J., McGordon A., Marco J. (2019). Cycle life of lithium ion batteries after flash cryogenic freezing. J. Energy Storage.

[7] Grandjean T.R., Groenewald J., Marco J. (2019). The experimental evaluation of lithium ion batteries after flash cryogenic freezing. J. Energy Storage.

[8] Hsieh A., Bhadra S., Hertzberg B., Gjeltema P., Goy A., Fleischer J.W., Steingart D.A. (2015). Electrochemical-acoustic time of flight: In operando correlation of physical dynamics with battery charge and health. Energy Environ. Sci..

[9] Davies G., Knehr K.W., Van Tassell B., Hodson T., Biswas S., Hsieh A.G., Steingart D.A. (2017). State of charge and state of health estimation using electrochemical acoustic time of flight analysis. J. Electrochem. Soc..

[10] Gold L., Bach T., Virsik W., Schmitt A., Müller J., Staab T.E., Sextl G. (2017). Probing lithium-ion batteries’ state-of-charge using ultrasonic transmission–Concept and laboratory testing. J. Power Sources.

[11] Kim J.Y., Jo J.H., Byeon J.W. (2020). Ultrasonic monitoring performance degradation of lithium ion battery. Microelectron. Reliab..

[12] Ladpli P., Kopsaftopoulos F., Chang F.K. (2018). Estimating state of charge and health of lithium-ion batteries with guided waves using built-in piezoelectric sensors/actuators. J. Power Sources.

[13] Zhao Y., Yang C., Yu Y. (2024). A review on covalent organic frameworks for rechargeable zinc-ion batteries. Chin. Chem. Lett..

[14] Soleimani Borujerdi A., Jin C., Zhu J. (2024). Ultrasonic monitoring of lithium-ion batteries with in-situ self-temperature correction. J. Power Sources.

[15] Owen R.E., Robinson J.B., Weaving J.S., Pham M.T.M., Tranter T.G., Neville T.P., Billson D., Braglia M., Stocker R., Tidblad A.A. (2022). Operando Ultrasonic Monitoring of Lithium-Ion Battery Temperature and Behaviour at Different Cycling Rates and under Drive Cycle Conditions. J. Electrochem. Soc..

[16] Sun H., Muralidharan N., Amin R., Rathod V., Ramuhalli P., Belharouak I. (2022). Ultrasonic nondestructive diagnosis of lithium-ion batteries with multiple frequencies. J. Power Sources.

[17] Chen C., Wei Y., Zhao Z., Zou Y., Luo D. (2019). Investigation of the swelling failure of lithium-ion battery packs at low temperatures using 2D/3D X-ray computed tomography. Electrochim. Acta.

[18] Qi Y., Hector L.G., James C., Kim K.J. (2014). Lithium concentration dependent elastic properties of battery electrode materials from first principles calculations. J. Electrochem. Soc..

[19] Swallow J.G., Woodford W.H., McGrogan F.P., Ferralis N., Chiang Y.M., Van Vliet K.J. (2014). Effect of electrochemical charging on elastoplastic properties and fracture toughness of Li_X_CoO_2_. J. Electrochem. Soc..

[20] Nitta N., Wu F., Lee J.T., Yushin G. (2015). Li-ion battery materials: Present and future. Mater. Today.

